# Evaluation of the Allelic Variations in Vernalisation (*VRN1*) and Photoperiod (*PPD1*) Genes and Genetic Diversity in a Spanish Spelt Wheat Collection

**DOI:** 10.3390/ijms242216041

**Published:** 2023-11-07

**Authors:** Carmen Palomino, Adoración Cabrera

**Affiliations:** Genetics Department, ETSIAM, Campus de Rabanales, Universidad de Córdoba, CeiA3, 14071 Córdoba, Spain; ge1pasam@uco.es

**Keywords:** wheat, spelt, vernalisation, photoperiod, flowering time, genetic diversity

## Abstract

Allelic variation within genes controlling the vernalisation requirement (*VRN1*) and photoperiod response (*PPD1*) determines the adaptation of wheat to different environmental growing conditions as well as influences other traits related to grain yield. This study aimed to screen a Spanish spelt wheat collection using gene-specific molecular markers for *VRN-A1*, *VRN-B1*, *VRN-D1*, and *PPD-D1* loci and to phenotype for heading date (HD) in both field and greenhouse experiments under a long photoperiod and without vernalisation. Fifty-five spelt genotypes (91.7%) exhibited a spring growth habit, and all of them carried at least one dominant *VRN1* allele, whereas five (8.3%) genotypes had a winter growth habit, and they carried the triple recessive allele combination. The *Vrn-D1s* was the most frequent allele in the studied set of spelt accessions, and it was found in combination with both the dominant *Vrn-A1b* and/or *Vrn-B1a* alleles in 88.3% of the spelt accessions tested. All spelt accessions carried the photoperiod-sensitive *Ppd-D1b* allele, which may explain the late heading of spelt germplasm compared to the commercial spring bread wheat Setenil used as a control. The least significant difference test showed significant differences between allelic combinations, the earliest accessions being those carrying two or three dominant alleles, followed by the one-gene combinations. In addition, the genetic diversity was evaluated through capillary electrophoresis using 15 wheat simple sequence repeat (SSR) markers. Most markers had high levels of polymorphism, producing 95 different alleles which ranged between 53 and 279 bp in size. Based on the polymorphic information content values obtained (from 0.51 to 0.97), 12 out of the 15 SSRs were catalogued as informative markers (values > 0.5). According to the dendrogram generated, the spelt accessions clustered as a separate group from the commercial bread wheat Setenil. Knowledge of *VRN1* and *PPD1* alleles, heading time, and genetic variability using SSR markers is valuable for spelt wheat breeding programs.

## 1. Introduction

Spelt wheat (*Triticum aestivum* ssp. *spelta* Thell., 2n = 6x = 42, A^u^A^u^BBDD) is an ancient wheat considered the ancestral form of common wheat (*T. aestivum* ssp. *aestivum,* 2n = 6x = 42, A^u^A^u^BBDD) which were originated in the Near East [1]. Spelt and common wheat are derived from a cross between emmer wheat (*Triticum turgidum* spp. *dicoccum* Schrank em. Thell., 2n = 4x = 28, A^u^A^u^BB), the first domesticated tetraploid wheat, and a wild grass (*Aegilops tauschii* Coss., 2n = 2x = 14, DD). Different origins of Asian and European spelt has been postulated. A secondary hybridisation between hexaploid wheat and emmer wheat has been suggested for the origin of European spelt [2,3]. In the past, spelt was widely cultivated, being an important food grain in Europe. At present, it is mainly grown in some regions of Germany, Austria, Belgium, and Switzerland. In Spain, the cultivated area is much smaller, and it is mostly used in organic farming [4]. Nonetheless, due to growing interest in natural foods in recent decades, the production of spelt has been boosted to satisfy rising consumer demand. Therefore, the cultivated area of spelt is increasing, and new spelt breeding programs have been started. The interest in spelt wheat is mainly associated with its use as a potential source of health-beneficial food [4,5]. However, spelt wheat has some agronomical problems such as lodging due to the high plant height, and its grain yield is lower than that of common wheat [6]. Thus, one of the objectives of spelt breeding programs is the obtention of shorter varieties, which allows for an increase in harvest index [7].

Spelt wheat is also considered a valuable source of new genes for wheat genetic improvement. This species has proved to be a rich reservoir of useful genes for tolerance to both biotic and abiotic stresses. For instance, accessions have been found with resistance to wheat yellow rust [8], leaf rust [9,10], or powdery mildew [10,11]. Genetic variation in spelt material in response to drought [12] and heat stresses [13] has been also identified, indicating that spelt may be used as a source of genes to strengthen the resistance of wheat lines to these stresses. Spelt germplasm has also shown variation in technological quality characteristics [14,15,16], and some studies have found that spelt grains contain a high content of essential amino acids as well as mineral ions such as those of zinc, magnesium, and iron, making it a good source of minerals and other nutrients [17,18,19].

Studies on phenology are of great importance, particularly in an era of increased climate variability. Genes controlling phenology play a central role in the adaptation of cereals to different climates as well as influence other traits related to grain yield [20,21]. Temperature and photoperiod are major environmental factors determining the transition to flowering in cereals. Vernalisation (*VRN*) and photoperiod (*PPD*) genes respond to both a lower temperature and a shorter day-length, respectively. There is detailed knowledge of the genetic control that determines vernalisation and photoperiod requirements in wheat and barley [22,23,24,25]. The *VRN* gene system controls the response of plants to low temperatures, exposure to which is necessary for the induction of flowering. The key gene that determines the vernalisation response in wheat is the MADS box transcription factor VERNALISATION1 (*VRN1*), which promotes the transition from vegetative to reproductive development [22,26,27]. Allelic variants of the *VRN1* genes are found on the long arm of chromosomes 5A, 5B, and 5D [28,29,30]. In common wheat, numerous alleles have been described at *VRN1* vernalisation loci. The different *VRN-A1* alleles (*Vrn-A1a*, *Vrn-A1b*, and *Vrn-A1c*) are the result of insertions and/or deletions in the promoter and intron-1 regions. Both the *VRN-B1* (*Vrn-B1a*, *Vrn-B1b*, and *Vrn-B1c*) and *VRN-D1* alleles (*Vrn-D1a*, and *Vrn-D1b*) mainly result from the deletion and/or insertion in the intron-1 region [31,32,33,34]. In *Triticum aestivum* ssp. *spelta*, a new allele (*Vrn-D1s*) was identified that is caused by DNA transposon insertion in the first intron [35]. One or more dominant alleles give rise to a spring growth habit (vernalisation insensitive), while a winter growth habit (vernalisation sensitive) is conferred by recessive alleles at the three loci [21].

On the other hand, a major gene controlling the day-length sensitivity of wheat is PHOTOPERIOD1 (*PPD1*), which encodes members of the pseudo-response regulator (PRR) family. *PPD1* loci are mapped on the short arm of chromosomes 2A, 2B, and 2D [36,37]. Among the three homoeologous genes, *PPD-D1* is considered the most important photoperiod regulator in wheat [36,38]. The photoperiod insensitivity conferred by the semi-dominant *Ppd-D1a* allele is caused by a large deletion upstream of the coding region [39,40]. The copy number variation for both the *VRN1* and *PPD1* genes has also been associated with the variation in vernalisation and photoperiod sensitivity of wheat, respectively [41].

The characterisation of the allelic variation phenology-related genes and the evaluation of the genetic diversity of spelt wheat germplasm would provide breeding programs with useful genetic resources necessary for the development of spelt varieties well adapted to local environmental growing conditions. The aims of this study were: (1) to assess the allelic variation in the *VRN-A1*, *VRN-B1*, *VRN-D1*, and *PPD-D1* genes in a collection of Spanish spelt wheat accessions using diagnostic molecular markers; (2) to examine the effect of allelic combinations on heading date under both greenhouse and field conditions; and (3) to evaluate genetic relationships within the spelt collection using SSR molecular markers. 

## 2. Results

### 2.1. Distribution Frequency of the VRN1 and PPD1 Alleles in the Spelt Collection

**Figure 1:**
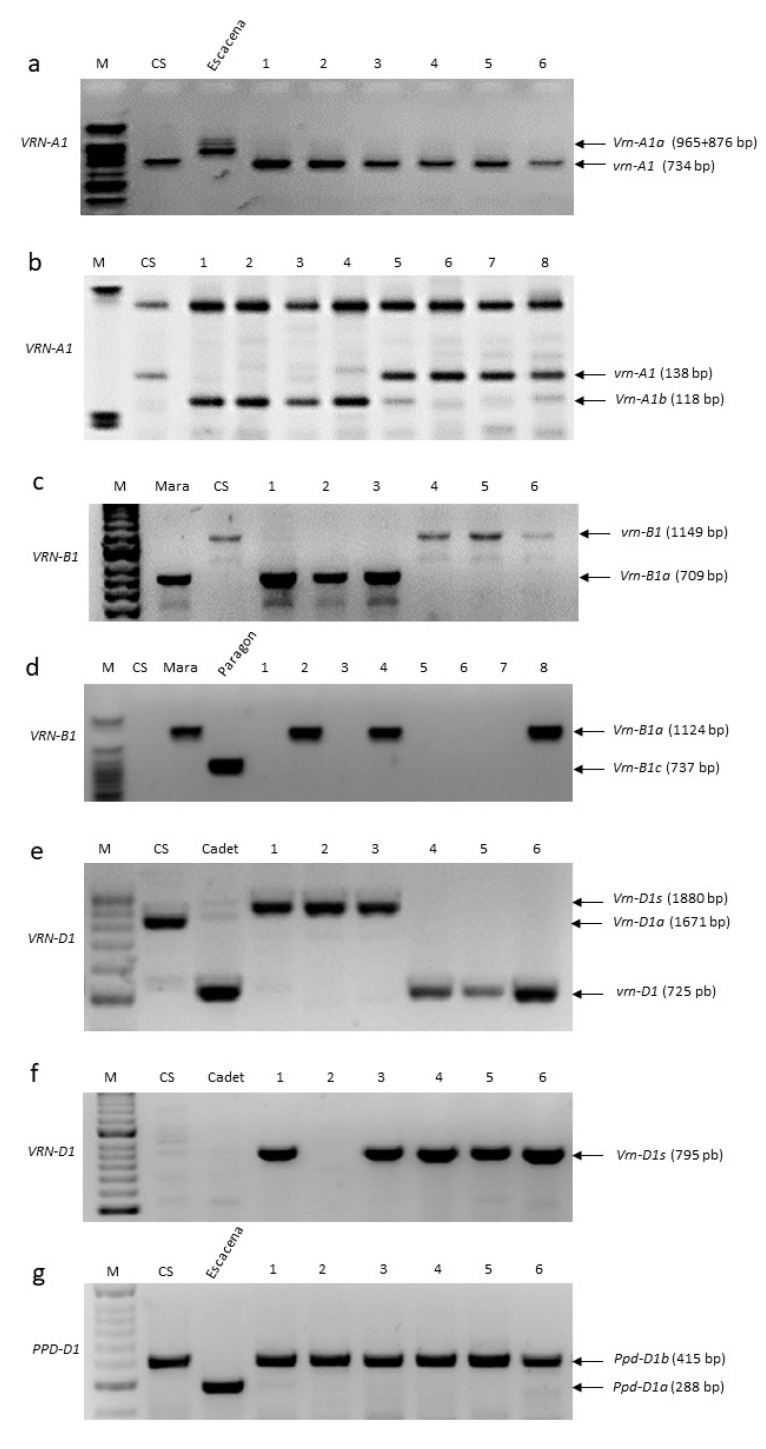
PCR amplification using allele-specific primers for *VRN-A1*, *VRN-B1*, *VRN-D1*, and *PPD-D1* loci of different spelt genotypes. (**a**) primers VRN1-AF and VRN1-INT1R to detect dominant *Vrn-A1a* and *Vrn-A1b* and recessive *vrn-A1* alleles of the *VRN-A1* gene; (**b**) MspI restriction patterns of the corresponding PCR products in Figure 1a, separated by polyacrylamide gels; (**c**) primers Intr1/B/F, Intr1/B/R3, and Intr1/B/R4 to detect *Vrn-B1a, Vrn-B1b*, and *vrn-B1* alleles of the *VRN-B1* gene; (**d**) primers Intr1 and Intr1/B/R3 to detect *Vrn-B1a* and *Vrn-B1c* alleles; (**e**) primers Intr1/D/F, Intr1/D/R3, and Intr1/D/R4 to detect *Vrn-D1a*, *Vrn-D1s*, and *vrn-D1* alleles of the *VRN-D1* gene; (**f**) Intr1/D/F and INSD-R primers specific to the *Vrn-D1s* allele; (**g**) primers Ppd-D1_F, Ppd-D1_R1, and Ppd-D1_R2 to detect dominant *Ppd-D1a* photoperiod-insensitive and recessive *Ppd-D1b* photoperiod-sensitive alleles of the *PPD-D1* gene. Common wheat cultivars Chinese Spring (CS), Mara, Cadet, Paragon, and Escacena were used as controls.

The results of the study of the alleles related to the flowering time genes are shown in Supplementary Table S1. To study allelic variation in the promoter region of the *VRN-A1* gene, we amplified genomic DNA using VRN1-AF and VRN1-INT1R primers as described by Yan et al. [31]. Two dominant alleles (*Vrn-A1a* and *Vrn-A1b*) and one recessive (*vrn-A1*) allele were identified by these primers. The amplification of genomic DNA using these primers yielded PCR products of 734 bp in length in all spelt accessions (Figure 1a), which may correspond to *Vrn-A1b* (714 bp) or *vrn-A1* (734 bp) alleles. To distinguish between these two alleles, the PCR product was digested with the MspI restriction endonuclease, and the fragments were separated using polyacrylamide gels. Two different restriction patterns were obtained: one 119-bp in length, which corresponds to the dominant *Vrn-A1b* allele with a 20 bp deletion in the promoter region, and the other 138 bp long which corresponds to the recessive *vrn-A1* allele (Figure 1b). 

A total of 51 (85.0%) spelt accessions carried the 119 bp-restriction fragment characteristic of *Vrn-A1b*, whereas 9 (15.0%) yielded the 138 bp fragment, indicating that they carried the recessive *vrn-A1* allele (Figure 1b). The common spring wheat Escacena variety has the foldback element insertion characteristic of the *Vrn-A1a* allele (Figure 1a). Neither the dominant promoter duplication allele *Vrn-A1a* nor the intron deletion allele *Vrn-A1c* were present in the spelt germplasm collection evaluated herein.

To identify allelic variation at the *VRN-B1* gene, multiplex PCR screening was carried out using three primer pairs (Intr1/B/F, Intr1/B/R3, and Intr1/B/R4). Three alleles could be identified with these primers, including two dominant (*Vrn-B1a* and *Vrn-B1b*) and one recessive (*vrn-B1*) [32]. Screening with these primers found two alleles in the spelt collection (Figure 1c). The dominant *Vrn-B1a* allele (709 bp) was carried by 13 (21.7%) spelt accessions, and the recessive *vrn-B1* allele (1149 bp) by 47 (78.3%) (Figure 1c). No spelt genotypes were found to carry the *Vrn-B1b* allele. Furthermore, the absence of the *Vrn-B1c* allele in the spelt collection was confirmed using the Intr1 and Int1/B/R3 primer combination (Figure 1d), as proposed by Shcherban et al. [42].

To investigate the distribution of the *VRN-D1* intron-1 allelic variation, a multiplex PCR was performed using three primer pairs: Intr1/D/F, Intr1/D/R3, and Intr1/D/R4. Electrophoretic separation of the PCR products identified intron-1 length polymorphisms resolving fragments of 997 bp (*vrn-D1*) and 1.841 bp (*Vrn-D1s*). The *vrn-D1* allele was identified in 7 (11.7%) accessions and *Vrn-D1s* in 53 (88.3%) accessions (Figure 1e). We did not find any genotypes containing the 1.670 bp fragment corresponding to the dominant *Vrn-D1a* allele in the spelt collection evaluated. A new reverse primer INSD-R was combined with the forward primer Intr1/D/F [32] and tested in this collection. Using these primer pairs specific for the *Vrn-D1s* allele, a 795 bp length amplicon was obtained, confirming the presence of this allele in the 53 spelt accessions (Figure 1f). In total, five accessions carried recessive alleles at the three vernalisation *VRN1* loci, and 55 carried at least one of the dominant vernalisation alleles tested. 

The PCR products obtained with the *PPD-D1*-specific primers in the spelt collection were all of the same size as the recessive *Ppd-D1b* (414 bp) photoperiod-sensitive allele from CS (Figure 1g), indicating that none of the spelt accessions has the dominant *Ppd-D1a* photoperiod-insensitive allele.

### 2.2. Effects of VRN1 Combinations on Spelt Heading Time 

Growth habit was assessed by growing the spelt accessions in both greenhouse and field conditions under a long photoperiod and without vernalisation. In accordance with Stelmakh et al. [20], the accessions that contained at least one dominant *VRN1* allele were classified as spring genotypes and those with three recessive alleles as winter genotypes. Out of the 60 spelt accessions tested, 5 (8.3%) failed to head under both greenhouse and field conditions, and all of them carried recessive (*vrn-A1*, *vrn-B1*, *vrn-D1*) vernalisation alleles at the three *VRN1* loci as identified by the PCR markers; these accessions were, therefore, classified as winter. Among these five accessions, one was from botanical var. *neglectum*, and two were from var. *arduii.* The other 55 (91.7%) accessions carried at least one of the dominant vernalisation alleles and all of them headed; hence, they were classified as spring (Supplementary Table S1).

Several different combinations of vernalisation alleles were found in the spelt collection. Table 1 shows the relationships between vernalisation and photoperiod genotypes and heading times under both greenhouse and field conditions. In total, there were five different types of genotypes grouped by allelic combination. A single dominant allele was observed only for *Vrn-A1b* (3.3%). We also observed two-dominant-allele combinations, namely, *Vrn-A1b—Vrn-D1s* (66.7%) and *Vrn-B1a—Vrn-D1s* (6.7%), and a three-dominant-allele combination, *Vrn-A1b*—*Vrn-B1a*—*Vrn-D1s* (15.0*%).* Finally, five (8.3%) accessions harboured triple-recessive *vrn-A1*—*vrn-B1*—*vrn-D1* alleles at the three loci.

**Figure 2:**
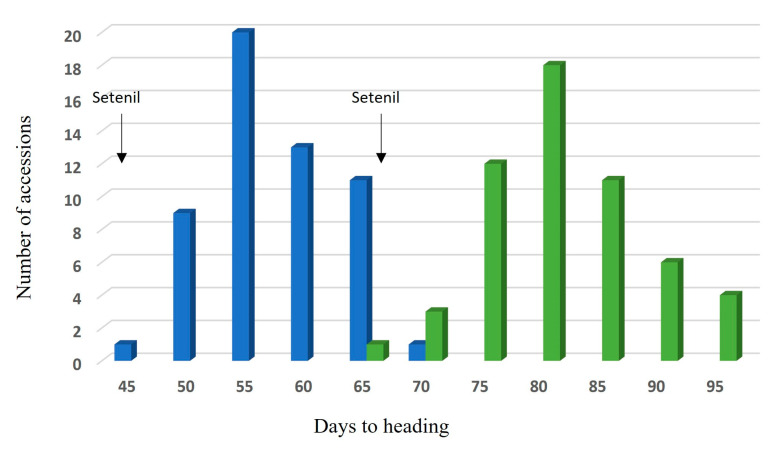
Distribution of spelt wheats by heading date under greenhouse (blue columns) and field (green columns) conditions, respectively.

The heading time recorded under both greenhouse and field conditions showed a normal distribution (Figure 2). The LSD tests revealed that the spring wheat Setenil cultivar used as a control, carrying the *vrn-A1*, *Vrn-B1a*, *Vrn-D1a*, *Ppd-D1a* allele combination, headed significantly earlier than the spelt accessions in both greenhouse and field experiments. Significant differences in the mean HD values were also found among the four spring spelt groups of allelic combinations. From field data, the LSD test showed that the spelt accessions carrying one dominant allele (*Vrn-A1b*) headed significantly later than those harbouring two (*Vrn-A1b*, *Vrn-D1s* or *Vrn-B1a*, *Vrn-D1s*) or three (*Vrn-A1b*, *Vrn-B1a*, *Vrn-D1s*) dominant alleles. No significant differences in the mean heading time were observed among genotypes carrying two or three allele combinations under field conditions. On the other hand, in greenhouse experiments, spelt genotypes carrying the triple dominant allele combination headed significantly earlier than both the genotypes carrying the double *Vrn-B1a*, *Vrn-D1s* combination and those with the single dominant allele *Vrn-A1b*. Furthermore, although genotypes carrying a single dominant *Vrn-A1b* allele headed later than those carrying two dominant alleles (*Vrn-A1b*, *Vrn-D1s* and *Vrn-B1a*, *Vrn-D1s*), the differences were not statistically significant under greenhouse conditions.

### 2.3. SSR and Cluster Analysis

Fifteen SSR markers were used to evaluate genetic variability in 60 accessions of spelt wheat. The parameters of variation analysed for SSR markers are presented in Table 2. Most SSRs showed high levels of polymorphism, generating a total of 95 different alleles with fragment sizes between 53 and 279 bp. The mean number of alleles per marker was 6.3, ranging from 1 to 19. Based on PIC values obtained (from 0.51 to 0.97, mean 0.71), 12 out of the 15 SSRs were classified as informative markers (PIC > 0.5). 

**Figure 3:**
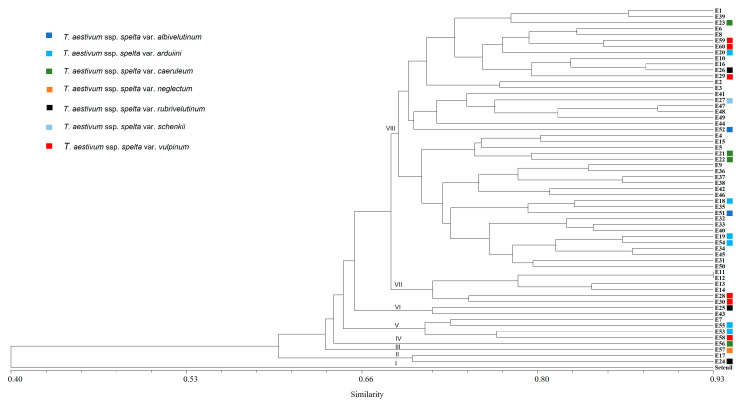
UPGMA dendrogram obtained from cluster analysis of 60 Spanish spelt wheat accessions based on the Dice similarity coefficient using 15 SSR markers.

The mean number of alleles per accession was 2.2, ranging from 1 to 5.8. SSR data were also used to establish genetic relationships among these accessions (Table 2). A dendrogram was obtained from the UPGMA analysis based on the Dice similarity coefficient (Figure 3). As would be expected, the dendrogram clustered the spelt wheat accessions as a separate group from the Setenil wheat variety. The 60 spelt accessions fell into seven main groups. Group II included two accessions, E17 and E24, while Groups III and IV were formed by just one accession, E57 and E56, respectively. Groups V, VI, and VII contained four (E7, E53, E55, and E58), two (E25 and E43), and six (E11, E12, E13, E14, E28, and E30) accessions, respectively. Finally, Group VIII was composed of the other 44 accessions (Figure 3).

Seven different botanical varieties were included in the spelt collection evaluated in this study (Supplementary Table S1). Among them, two (E51 and E52) were from var. *albivelutinum*, six (E18, E19, E20, E53, E54, and E55) from var. *arduii*, four (E21, E22, E23, and E56) from var. *caeruleum*, one (E57) from var. *neglectum*, three (E24, E25, and E26) from var. *rubrivelutinum*, one (E27) from var. *schenkii*, and six (E28, E29, E30, E58, E59, and E60) from var. *vulpinum* (Supplementary Table S1). The dendrogram obtained showed that these 23 accessions belonging to the seven spelt botanical varieties did not cluster separately; rather, these accessions were scattered across all spelt Groups in the dendrogram (Figure 3).

## 3. Discussion

In the present study, we used specific DNA markers to assess the allelic variation at the *VRN-A1*, *VRN-B1*, *VRN-D1*, and *PPD-D1* genes in a collection of spelt wheat accessions, and we examined the effect of their allelic combinations on heading date under both field and greenhouse conditions.

Within the spelt collection, the highest allele frequency was recorded for the dominant *Vrn-D1s* allele which was found in 53 (88.3%) accessions. The second most common was the dominant *Vrn-A1b* allele, detected in 51 (85.0%) accessions, followed by the recessive *vrn-B1*, detected in 48 (80.0%) accessions.

The *Vrn-A1a* allele has been found to be the dominant allele in common spring wheat cultivars in many regions of the world [31,43,44,45]. In this study, we did not find the dominant *Vrn-A1a* allele in any of the spelt accessions evaluated. We found that all the spelt wheat genotypes carrying the dominant *VRN-A1* gene yielded the 118 bp specific restriction pattern with the MspI enzyme corresponding to the *Vrn-A1b* allele. The *Vrn-A1b* allele has been found in both tetra- and hexaploid wheat accessions [31,44,45,46], but it has not been found in diploid wheat species [31,47]. The *Vrn-A1b* allele has also been found in spelt genotypes from diverse origins [48,49] and is common in bread wheat cultivars from Palestine [49] and Spain, Portugal, Italy, and Greece [50]. In relation to the *VRN-B1* gene, our results showed that the recessive *vrn-B1* allele was the most prevalent, being present in 80% of the accessions examined, followed by the dominant *Vrn-B1a* allele (in 20% of the accessions). In common wheat *T. aestivum*, research has found the *Vrn-B1a* allele to be the most prevalent, followed by the recessive allele, while *Vrn-B1b* was less common [51]. The *Vrn-B1b* and *Vrn-B1c* alleles have also been found in spelt accessions from different origins [48,51]. In contrast, no spelt genotypes carrying the *Vrn-B1b* or *Vrn-B1c* alleles were found in our study.

The *Vrn-D1s* intron-1 insertion was identified by Muterko et al. [35] and represents a member of a new transposon family. These authors found that the *Vrn-D1s* allele was present in three spelt accessions from Spain and England and one *T. compactum* accession. In this study, the *Vrn-D1s* allele was found in 88.3% of spelt accessions and was the dominant allele found in the Spanish spelt collection. Analysing spelt accessions from various origins, Dragovich et al. [49] also found that accessions from Spain carried the *Vrn-D1s* allele. On the other hand, only one accession carrying the *Vrn-D1s* allele was identified by Curzon et al. [48] in evaluating a wide European spelt collection, the recessive allele *vrn-D1s* being the most frequently detected at the *VRN-D1* locus in the panel of European spelt wheat evaluated by these authors. All these results show that the *Vrn-D1s* allele is more frequently found in Spanish spelt than in spelt from Central Europe. European spelt accessions have been classified into two eco-geographical groups, Iberian (pol. *ibericum* Flaskb.) and Bavarian (pol. *bavaricum* Vav.), with the latter including accessions from Germany, Belgium, Switzerland, and Eastern Europe, and the distinct differences between these groups indicated that they have evolved independently for a long time [52]. The high frequency of the *Vrn-D1s* allelic variant found in the Spanish spelt collection evaluated here and the scarceness of this allele in European spelt accessions [48] support the hypothesis that Iberian spelt constitutes a distinct geographical group differing from the rest of European spelt [4,49,52]. Spelt germplasm from Spain has been also considered a separate gene pool within the spelt germplasm based on analysis using SSR markers [53].

Based on the heading date of the spelt wheat accessions in the present study, most genotypes (91.7%) had a spring seasonal growth habit, with only five (8.3%) genotypes exhibiting a winter growth habit. PCR using gene-specific molecular markers demonstrated the presence of at least one dominant *VRN1* allele in all the spring genotypes, while accessions carrying the triple recessive allele combination did not form ears under any greenhouse or field conditions with a long photoperiod and without vernalisation. These results showed consistency between vernalisation alleles and spelt growth habit.

The spelt wheat collection exhibited late heading compared to the local spring wheat cultivar control Setenil (Figure 2, Table 1). The Setenil cultivar carried the two dominant *Vrn-B1a* and *Vrn-D1a* alleles and the photoperiod-insensitive allele *Ppd-D1a* (Figure 1g). In contrast, PCR screening of the *PPD-D1* gene showed that all spelt accessions amplified the 414 bp fragment characteristics of genotypes with the *Ppd-D1b* photoperiod-sensitive allele. The presence of the photoperiod-insensitive allele *Ppd-D1a* in the bread wheat Setenil may explain the earlier heading time shown by this cultivar compared with the spelt accessions. The screening of *PPD1* alleles in spelt germplasm by other authors [48,49] has also shown the prevalence of the *Ppd-D1b* allele in spelt genotypes, and all late heading accessions have been found to carry the photoperiod-sensitive allele.

The LSD test revealed significant differences in the mean values of the HD among the four spring spelt genotypes, indicating that the *VRN1* allele combinations were also influencing the differences in HD observed. According to the LSD results, spelt genotypes carrying at least one dominant *VRN1* allele (*Vrn-A1b*, *vrn-B1*, *vrn-D1*) headed significantly later than those harbouring two (*Vrn-A1b, Vrn-D1s* or *Vrn-B1a, Vrn-D1s*) or three (*Vrn-A1b, Vrn-B1a, Vrn-D1s*) dominant alleles in both greenhouse and field experiments. However, only two spelt accessions carried the *Vrn-A1b*, *vrn-B1*, and *vrn-D1* alleles; therefore, the sample size was so small that we cannot rule out the possibility that the LSD significant data are not statistically representative.

Muterko et al. [35] classified the spelt accessions carrying the *Vrn-D1s* allele as spring wheat based on passport data. In this study, this allele was found in combination with the dominant *Vrn-A1b* and/or *Vrn-B1a* alleles in 88.3% of the spelt accessions tested. The influence of *Vrn-D1s* on the spring growth habit of spelt accessions cannot be determined with certainty due to the presence of these other dominant alleles in the genotypes evaluated. Nonetheless, all the accessions carrying the *Vrn-D1s* allele headed under long-day conditions without vernalisation in both greenhouse and field experiments, indicating that they had a spring growth habit. Other allelic forms have been detected in the *VRN1* genes in wheat [35,54,55], and we cannot rule out these influencing the phenology of spelt lines. These forms include the duplication of the *VRN-B1* locus observed recently in some accessions of *T. aestivum* ssp. *spelta* and *T. compactum* [56], as well as copy number variations in both recessive and dominant *VRN1* alleles reported in bread wheat [41,56,57]. Polymorphism in exons 4 and 7 of the *VRN-A1* gene has also been detected in different wheat species [55].

The objective of this study was to assess genetic variability in the spelt accessions using SSR markers and capillary electrophoresis. A high level of polymorphism was found in the spelt collection (PIC > 0.5). The most informative marker was *Xgwm397*, with a total of 13 alleles and a PIC value of 0.97 (Table 2). In the germplasm collection evaluated, the mean number of alleles per molecular marker was 6.3, which is higher than that found in previous studies in European spelt cultivars where the average was 5.18 per marker [58]. Similarly, the average PIC value calculated in this study was 0.71, which is also higher than that found previously in spelt materials [53,58].

Previous studies on genetic diversity using molecular markers have found that spelt accessions clustered as a separate group from other hexaploid species, including common wheat [53,59,60,61]. The dendrogram obtained in this study was consistent with these previous studies and clearly separated the spelt accessions from the common wheat Setenil. Furthermore, spelt accessions from Spain were clustered in different groups to those from Germany and Belgium [59], supporting the hypothesis that Spanish spelt constitutes a separate gene pool within the spelt germplasm [49,52,53,54].

Twenty-three accessions belonging to the seven spelt botanical varieties were included in the Spanish collection evaluated in this study (var. *albivelutinum*, var. *arduii*, var. *caeruleum*, var. *neglectum*, var. *rubrivelutinum*, var. *schenkii*, and var. *vulpinum* (Supplementary Table S1)). Except for those from var. *neglectum* which clustered separately in Group III, the accessions (n = 22) were scattered across the dendrogram obtained, indicating that these botanical varieties cannot be distinguished with the SSR markers used. Accessions with identical SSR patterns were found, i.e., E11 and E12, which clustered together in the dendrogram, and they also have an identical allelic composition at both the *VRN1* and *PPD-D1* loci (Supplementary Table S1). These results indicated that these accessions were probably duplicated and that the SSR analysis can also help to identify duplicate accessions in germplasm collections.

Variation in spelt phenology is important for crop adaptation. Due to the growing interest in spelt wheat and the increase in the cultivated area, variation in phenology is important for the cultivation of spelt across new or broader geographical ranges, suited to local growing conditions.

## 4. Material and Methods

### 4.1. Plant Material

A collection of 60 spelt wheat accessions was employed in this study. Seeds were supplied by the Plant Genetic Resources Centre (CRF) at the National Institute for Agricultural Research (INIA, Alcalá de Henares, Spain). Seven botanical varieties were included in the spelt collection evaluated. Two were from var. *albivelutinum*, six from var. *arduii*, four from var. *caeruleum*, one from var. *neglectum*, three from var. *rubrivelutinum*, one from var. *schenkii*, and six from var. *vulpinum*. Accession numbers of the spelt collection are given in Supplementary Table S1. Spring wheat cultivars, namely, Chinese Spring (CS), Mara, Cadet, Paragon, Escacena, and Setenil, were used as control.

### 4.2. Evaluation of the Heading Time

Field and greenhouse experiments were carried out in the 2021–2022 growing season in Córdoba, south of Spain. For each accession, three germinated seeds were sown individually in plastic pots and grown in a greenhouse at 20–25 °C under long-day conditions (18 h day-length) without vernalisation. The heading time was recorded as the time that the first spike emerged from the flag leaf on the main stem, and the mean was calculated from the three plants grown per accession. For evaluation of the ear emergence time of spelt lines in the field, the seeds were sown on 1 March 2022 in single rows (150 cm length) at a row spacing of 40 cm row in an experimental field of the University of Córdoba (Spain) without vernalisation and under a long-day photoperiod. In this case, the heading date was recorded when the first spike emerged from the flag leaf sheath in half of the plants for each accession. Days to heading (DH) was calculated as the number of days from sowing to heading. DH values were recorded twice weekly in both the greenhouse and field conditions. The local spring wheat cultivar Setenil was included as a control. To evaluate the statistical significance of differences in heading time between the spelt groups of allelic combinations, analysis of the variance was carried out, and mean values were compared using the least significant difference (LSD) test (*p* < 0.05). Statistical analysis was performed with Statistix version 8.0.

### 4.3. DNA Markers Analysis

Total genomic DNA was extracted from frozen young (4-leaf stage) plants using the cetyltrimethylammonium bromide method [62]. To prepare DNA samples for each accession, equal amounts of leaves from three individual plants of each accession were bulked. Samples were stored at −20 °C until amplification through PCR. The concentration of DNA in each sample was estimated using a Nano-Drop 1000 Spectrophotometer (Thermo Scientific, Waltham, MA, USA).

### 4.4. PCR Markers to Genotype the Spelt Wheat Collection for VRN1 and PPD1 Loci

To determine the alleles of the *VRN-A1*, *VRN-B1*, *VRN-D1*, and *PPD-D1* homoeologous genes, we used gene-specific molecular markers reported in previous studies (Supplementary Table S2). Allelic variants of the *VRN-A1* gene were identified using the primer pair VRN1AF and VRN1-INTR1R, which is associated with the promoter region and allows for the detection of the dominant (*Vrn-A1a* and *Vrn-A1b*) and recessive (*vrn-A1*) alleles [31]. To distinguish between *Vrn-A1b* and *vrn-A1* alleles, the PCR product was digested with restriction endonuclease MspI as in Shcherban et al. [42], and DNA fragments were separated on polyacrylamide gels. The allele variants of the *VRN-B1* gene were evaluated using three gene-specific primers (Intr1/B/F, Intr1/B/R3, and Intr1/B/R4), a combination linked to the intron-1 in a multiplex PCR reaction, which allows for *Vrn-B1a*, *Vrn-B1b*, and *vrn-B1* alleles to be detected [32,33]. Primers Intr1 and Intr1/B/R3 were used to differentiate between *Vrn-B1a* and *Vrn-B1c* as described by Shcherban et al. [42]. Similarly, to study the distribution of *VRN-D1* intron-1 allelic variation, multiplex PCR was performed using three primer pairs (Intr1/D/F, Intr1/D/R3, and Intr1/D/R4). This PCR makes it possible to distinguish between *Vrn-D1a, Vrn-D1s*, and *vrn-D1* allelic variants [32,35]. Additionally, a new primer INSD-R was designed in this study using the publicly available *Vrn-D1s* gene sequence (GenBank accession KF800714) from *T. aestivum* ssp. *spelta* strain PI 348700 [35] and primer 3 software (http://frodo.wi.mit.edu/primer3/) (accessed on 3 October 2023) [63]. Using the primer pairs Intr1/D/F and INSD-R, a 795 bp length amplicon is expected for the detection of the *Vrn-D1s* allele.

Further, to identify allelic variants of the *PPD-D1* gene, three specific primers (Ppd-D1-F1, Ppd-D1-R1, and Ppd-D1-R2) were utilised in a multiplex PCR assay, allowing the photoperiod-insensitive and -sensitive alleles to be distinguished. Primer pair Ppd-D1-F and Ppd-D1-R2 produced a 288 bp fragment in genotypes with the photoperiod-insensitive allele *Ppd-D1a*, whereas primer pair Ppd-D1-F and Ppd-D1-R1 yielded a 414 bp fragment in genotypes with the photoperiod-sensitive allele *Ppd-D1b* [39].

PCR was performed with 40 ng of template DNA in a 25 μL volume reaction mixture containing 5 μL of 1× PCR buffer, 0.4 µM of each primer, 1.5 mM of MgCl_2_, 0.4 mM of dNTPs, and 0.25U of Taq DNA polymerase (Promega, Madison, WI, USA). PCR conditions were as follows: 4 min at 94 °C, followed by 35 cycles of denaturation at 94 °C for 45 s, annealing for 45 s, extension at 72 °C for 1 min, and then a final extension at 72 °C for 5 min. Amplified products were resolved on 2.0% agarose gels or separated by polyacrylamide gels (10% *w*/*v*, C: 2.67%) stained with ethidium bromide. Primer sequences used in this study and annealing temperatures for each primer combination are listed in Supplementary Table S2.

### 4.5. SSR Analysis and Genetic Variation

Fifteen primer pairs developed in wheat (*Triticum aestivum* L.) by Röder et al. [64] were selected and employed to evaluate genetic variation in 60 accessions of spelt wheat. The commercial hexaploid wheat variety Setenil was included in the analysis. PCR amplification (final volume: 10 µL) consisted of 20 ng of genomic DNA, 2 mM of MgCl_2_, 0.8 mM of dNTP, 5X polymerase buffer, 0.125 µM of forward primer, 0.25 µM of reverse primer, 0.25 µM of fluorochromes (FAM or 6-HEX), and 1 U of Taq polymerase (Promega). Forward primers were synthesised with a 19 bp long M13 tail (5′-CACGACGTTGTAAAACGAC-3′). The cycling protocol was: 1 min at 94 °C, 35 cycles at 94 °C for 1 min, annealing at 53 °C for 1 min, and polymerisation at 72 °C for 1.5 min, followed by a final extension at 72 °C for 10 min. The amplification products were separated using an automated capillary sequencer (ABI 3130 Genetic Analyzer: Applied Biosystems/HITACHI, Madrid, Spain) in the Genomics Unit of the Central Research Support Service at the University of Córdoba. The size of the amplified bands was determined based on an internal DNA standard (400HDROX) with GeneScan software (version 3.x). The results were interpreted using Genotyper software (Version 3.7), all from Applied Biosystems. The number of different alleles and polymorphic information content (PIC) were also determined [65]. The Dice coefficient was calculated as a measure of similarity between accessions. Cluster analysis was performed using the unweighted pair group method with arithmetic means (UPGMA), a hierarchic agglomerative method equivalent to the average linkage between groups method. A dendrogram was calculated using the Numerical Taxonomy and Multivariate Analysis System software NTSYS Version 2.0 (Applied Biostatistics, Setauket, NJ, USA).

## 5. Conclusions

Allelic profiles at the *VRN1* and *PPD-D1* loci of the Spanish spelt accessions and their association with heading time in both field and greenhouse experiments indicated that most accessions have a spring seasonal growth habit, but they were photoperiod-sensitive (*Ppd-D1b*), and this could explain another observation, namely, the late heading of spelt accessions compared with that of the local Setenil wheat cultivar. PCR using gene-specific molecular markers demonstrated the presence of at least one dominant *VRN1* allele in all the spring genotypes while accessions carrying the triple recessive allele combination did not flower under any greenhouse and field conditions under a long photoperiod and without vernalisation. The two spring alleles, *Vrn-A1b* and *Vrn-D1s*, were the most frequent, and the *Vrn-A1b*, *vrn-B1*, *Vrn-D1s* allelic combination was the most characteristic of the Spanish spelt collection. A high polymorphic information content value was calculated using SSRs in the spelt collection, and the dendrogram clustered the spelt germplasm as a separate group from the wheat Setenil variety.

## Figures and Tables

**Table 1:** Mean number of days ± SE from sowing to heading for *VRN1* and *PPD-D1* allele combinations in the Spanish wheat spelt collection.

	Allelic Composition			Nº of Accessions (%)	Average Heading Time (Nº of Days to Heading ± SE)	
					Greenhouse	Field
^1^ *vrn-A1*	***Vrn-B1a***	***Vrn-D1a***	***Ppd-D1a***	-	49.0 a	63.0 a
***Vrn-A1b***	***Vrn-B1a***	***Vrn-D1s***	*Ppd-D1b*	9 (15.0%)	59.0 ± 1.9 b	79.7 ± 2.5 b
***Vrn-A1b***	*vrn-B1*	***Vrn-D1s***	*Ppd-D1b*	40 (66.7%)	59.3 ± 0.8 bc	83.6 ± 0.9 b
*vrn-A1*	***Vrn-B1a***	***Vrn-D1s***	*Ppd-D1b*	4 (6.7%)	65.2 ± 1.8 c	79.8 ± 2.4 b
***Vrn-A1b***	*vrn-B1*	*vrn-D1*	*Ppd-D1b*	2 (3.3%)	67.0 ± 1.0 c	95.5 ± 1.4 c
*vrn-A1*	*vrn-B1*	*vrn-D1*	*Ppd-D1b*	5 (8.3%)	No spikes	No spikes

^1^ Bread wheat Setenil cultivar used as control. Means with the same letters in the same column are not significantly different for an LSD test at *p* < 0.05. Dominant alleles are in bold.

**Table 2:** Marker size range, number of alleles, and polymorphic information content (PIC) observed in 60 accessions of the Spanish spelt wheat collection studied with 15 microsatellite markers.

SSR Marker	Size Range (bp)	Number of Alleles		PIC ^c^
		N ^a^	Mean ^b^	
*Xgwm44*	53	1	1	-
*Xgwm135*	133	1	1	-
*Xgwm219*	152–210	8	2.5	0.77
*Xgwm257*	192–213	3	1.5	0.64
*Xgwm285*	102–259	8	1.9	0.75
*Xgwm291*	120–133	4	2.0	0.54
*Xgwm294*	64–100	3	1.9	0.54
*Xgwm314*	111–139	2	1.7	0.51
*Xgwm382*	104–175	19	2.1	0.91
*Xgwm397*	190–279	13	5.8	0.97
*Xgwm403*	75–128	4	3.7	0.75
*Xgwm513*	158–164	4	1.2	0.58
*Xgwm544*	207–239	11	2.1	0.72
*Xgwm565*	86	1	1	-
*Xgwm614*	139–174	13	3.1	0.88
Total		95	-	-
Mean		6,3	2.2	0.71

^a^ Number of different alleles. ^b^ Mean number of alleles per accession. ^c^ Calculated on polymorphic markers.

## Data Availability

Not applicable.

## Supplementary Materials

The following supporting information can be downloaded at: https://www.mdpi.com/article/10.3390/ijms242216041/s1.
